# Regulation of Mitochondrial Homeostasis and Nrf2 in Kidney Disease: Timing Is Critical

**DOI:** 10.1155/2022/9275056

**Published:** 2022-04-28

**Authors:** Yuxian Zhuang, Liue Hu, Yang Wu, Chen Yang, Shangmei Li, Kaipeng Jing, Huafeng Liu

**Affiliations:** ^1^Guangdong Medical University, Zhanjiang, 524023 Guangdong, China; ^2^Affiliated Hospital of Guangdong Medical University, Zhanjiang, 524001 Guangdong, China; ^3^Key Laboratory of Prevention and Management of Chronic Kidney Disease of Zhanjiang City, Zhanjiang, 524001 Guangdong, China

## Abstract

Abnormal regulation of mitochondrial homeostasis plays a critical role in the progression of renal disease. Recent studies have shown that activation of nuclear factor erythroid 2-related factor 2 (Nrf2) has time-dependent protective effects, which can be explained by the differing regulation of mitochondrial homeostasis during the various stages of kidney disease. In this review, we summarize the mechanisms whereby mitochondrial homeostasis is regulated and the nature of the dysregulation of mitochondrial homeostasis in renal disease. In addition, we summarize the dual roles of Nrf2 in kidney disease by discussing the studies that have shown the importance of the timing of its activation in the regulation of mitochondrial homeostasis. This should provide a theoretical basis for therapeutic strategies aimed at activating Nrf2 in kidney disease.

## 1. Introduction

The spectrum of kidney diseases includes acute kidney injury (AKI) and chronic kidney disease (CKD), which can have a variety of etiologies. AKI is defined as renal dysfunction that develops over a very short period of time and is characterized by a rapid increase in circulating creatinine concentration, low urine output, and an impairment in fluid balance. Because of a lack of specific therapies, it is associated with a poor prognosis and high mortality in the short term, and it also increases the risks of CKD and death in the long term [1]. CKD is defined as a kidney disease in which kidney function is abnormal or there is damage to kidney tissue over a period of >3 months and that progresses to end-stage renal disease (ESRD) and death [2]. To develop more effective treatments, it is essential to understand the pathogenesis of these diseases in more detail.

Kidney function is highly dependent on the presence of plentiful mitochondria, which mediate rapid metabolism, involving vast oxygen consumption. Therefore, mitochondrial injury plays a substantial role in the pathogenesis of kidney disease [3]. Mitochondrial injury occurs secondary to abnormal regulation of mitochondrial homeostasis, which requires the coordinated regulation of several opposing processes, including reduction-oxidation (redox) reactions, mitochondrial fission and fusion, and mitophagy and mitochondrial biogenesis. These processes may be beneficial or detrimental at particular stages of a disease, depending on the local environment. For example, during the early stages of AKI, the regulation of mitochondrial dynamics plays a decisive role in damage repair [4], whereas an upregulation of mitochondrial biogenesis may cause further damage [5]. Furthermore, mitochondrial biogenesis may facilitate repair and prevent the progression of AKI to CKD during the later stage of AKI [6] but may be deleterious in CKD because it promotes renal fibrosis [7].

**Figure 1:**
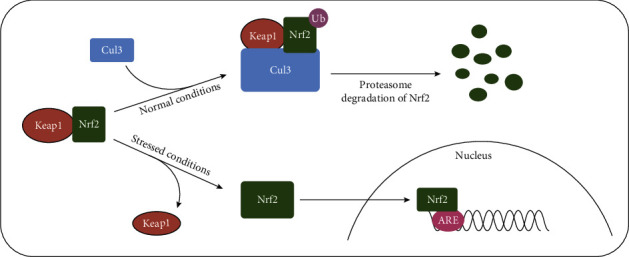
Schematic diagram of Nrf2 activity regulated by Keap1 under different conditions. Under normal conditions, Keap1 connects with Nrf2 and Cullin3 (Cul3) to form a Keap1/Nrf2/Cul3 complex. Subsequently, Cul3 mediates the ubiquitination and proteasomal degradation of Nrf2. Under stressed conditions, Keap1 undergoes a conformational change and releases Nrf2 from the complex, thus leading to the translocation of Nrf2 into the nucleus and increase of the transcription of antioxidant genes by binding to the ARE.

As an upstream regulator of mitochondrial homeostasis, nuclear factor erythroid 2-related factor 2 (Nrf2) has a regulatory role in kidney disease. Kelch-like ECH-associated protein 1 (Keap1) is responsible for the negative regulation of Nrf2. Under physiological conditions, it acts as an adaptor protein for Cullin3 in Cullin-RING E3 ubiquitin ligase complexes, mediating the binding of Cullin3 to its target protein Nrf2, which leads to the latter's ubiquitination and subsequent proteasomal degradation. However, under stress conditions, Keap1 is selectively oxidized by oxidative/electrophilic stimulants, which promotes conformational changes and the release of Nrf2, leading to the expression of antioxidant genes via the binding of Nrf2 to antioxidant responsive elements (ARE) [8] (Figure 1). Previous experimental studies have shown that Nrf2 inactivation is essential for the progression of kidney disease, whereas its genetic or pharmacological activation attenuates kidney damage and prevents some adverse outcomes. However, the use of the Nrf2 activator bardoxolone methyl for the treatment of diabetic nephropathy was associated with the serious complications of heart failure and fluid retention in a clinical trial, which led to its premature termination, and suggests that Nrf2 activation in CKD may have detrimental effects [9]. Furthermore, several studies of AKI have indicated that the activation of Nrf2 is more beneficial when it occurs early in the course of the disease. Thus, despite the efficacy and safety of strategies aimed at the early activation of Nrf2 remaining unclear, the discovery that the effects of Nrf2 depend on the timing of activation has guided subsequent research and the development of treatment strategies.

In the present review, we aim to clarify the dual effects and actual value of Nrf2 activation in kidney disease by summarizing the beneficial and deleterious effects of Nrf2 activation in kidney disease, according to its effects on mitochondrial homeostasis and its timing. Because ischemia/reperfusion-induced, sepsis-associated, and drug-induced AKI, as well as diabetes-associated CKD, are common manifestations of kidney disease that are usually associated with poor prognosis, and the potential protective effects of Nrf2 for these diseases have been thoroughly investigated in preclinical studies or clinical trials, we focus on these specific types of kidney injury.

## 2. Mechanisms of Mitochondrial Homeostasis

### 2.1. Redox Reactions

**Figure 2:**
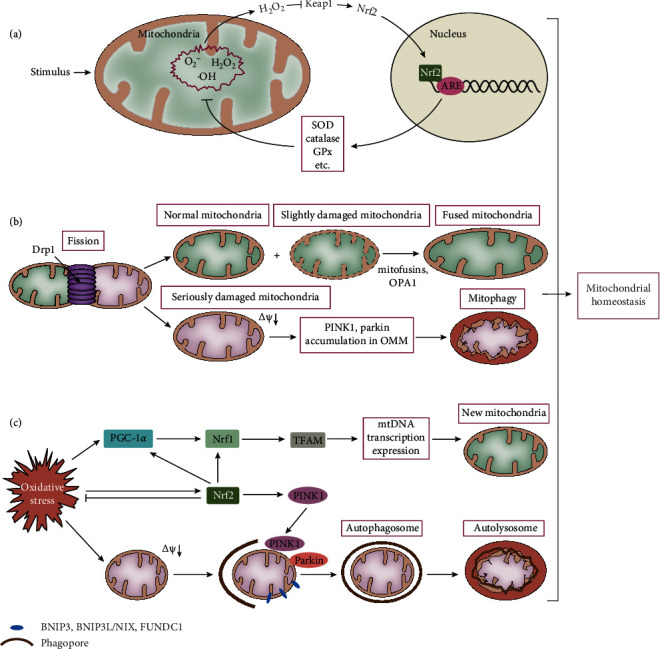
Three mechanisms are involved in the regulation of mitochondrial homeostasis: redox status, mitochondrial dynamics, and mitophagy and mitochondrial biogenesis. (a) Redox: under stress conditions, mitochondria generate a massive amount of ROS, which are mainly composed of the O_2_^−^, H_2_O_2_, and •OH species. H_2_O_2_ acts as a signaling molecule that inhibits cytoplasmic Keap1, resulting in the transfer of Nrf2 to the nucleus, where it increases the transcription of antioxidant genes, such as those expressing SOD, GPx, and catalase, by binding to ARE in their promoters. (b) Mitochondrial dynamics: damaged mitochondria are split into normal mitochondria and severely damaged mitochondria, via a process mediated by Drp1. Normal mitochondria and other slightly damaged mitochondria fuse to form intact mitochondria, via a process mediated by mitofusins and OPA1, while severely damaged mitochondria undergo mitophagy induced by the aggregation of PINK1 and Parkin in the OMM following a decrease in membrane potential. (c) Mitophagy and mitochondrial biogenesis: under oxidative stress conditions, the injured mitochondria bind to the phagophores indirectly via the PINK1/Parkin pathway or directly via outer membrane protein receptors (BNIP3, BNIP3L/NIX, and FUNDC1), leading to the formation of autophagosomes and autolysosomes, thus leading to their removal by lysosomes. The activation of the PGC-1*α*/Nrf1/TFAM pathway induces mtDNA transcription and the production of new mitochondria. Nrf2 not only increases mitophagy by promoting the expression of PINK1 but also facilitates mitochondrial biogenesis by promoting the expression of PGC-1*α* and Nrf1. Therefore, Nrf2 acts as a link between mitophagy and mitochondrial biogenesis.

Under normal circumstances, mitochondria generate reactive oxygen species (ROS) as byproducts during the production of adenosine triphosphate (ATP) by aerobic respiration. To prevent cellular damage owing to an accumulation of ROS, excessive amounts of ROS are removed by antioxidant reactions. However, during states of stress and disease, particular stimulants cause rapid increases in ROS concentrations, which lead to oxidative stress and damage. The main types of ROS are the superoxide radical (O_2_^−^), hydrogen peroxide (H_2_O_2_), and the hydroxyl radical (•OH) [10], and to prevent oxidative damage, a sophisticated antioxidant system has evolved that has a number of components, including superoxide dismutase enzymes (SODs), glutathione peroxidase (GPx), catalase, and reduced glutathione [11]. Although ROS are widely appreciated to cause damage, their roles in normal signal transduction have recently also been studied. In fact, H_2_O_2_ is considered to be the most important signaling molecule for the regulation of redox homeostasis because it is not a free radical, meaning that it is stable and can diffuse readily [12]. The best-known effector of H_2_O_2_ is Keap1, which is oxidized at the sulfhydryl group of active L-cysteine by H_2_O_2_ [12]. This thiol oxidation leads to a conformational change in Keap1 and the release of Nrf2, which increases the stability and activity of Nrf2, thereby increasing the transcription of antioxidant genes, such as those expressing SOD, GPx, and catalase [13] (Figure 2(a)).

### 2.2. Mitochondrial Dynamics

Mitochondria in living cells typically have long tubular morphology, with interconnections that create a branching network that is in a highly dynamic state of fission and fusion [14]. Highly variable mitochondrial networks are thought to mediate adaptations of the body to environmental stimuli, which help to coordinate energy requirements in response to disturbances in energy supply. The coordinated regulation of mitochondrial fission and fusion, also known as mitochondrial dynamics, is a fundamental mechanism for the maintenance of a complete and healthy mitochondrial network. Mitochondrial fission is the process by which mitochondria split in two, and this is principally mediated by dynamin-related protein 1 (Drp1) [15] (Figure 2(b)). Under normal conditions, mitochondrial fission helps to promote the detachment of damaged or aged mitochondria from the mitochondrial network, and the detached abnormal mitochondria are removed from the cytoplasm by mitophagy, which ensures that the mitochondrial network remains healthy [16] (Figure 2(b)). However, mitochondrial fission also represents a means of increasing the number of mitochondria and widening their spatial distribution [17]. The opposite process of mitochondrial fusion is the fusion of two mitochondria to form a single mitochondrion, which involves mitofusin- (Mfn1/2-) mediated outer membrane fusion and optic atrophy 1- (OPA1-) mediated inner membrane fusion [15] (Figure 2(b)). Mitochondrial fusion is required to maintain the normal tubular morphology of mitochondria, which is the structure associated with optimal energy generation [18]. In addition, slightly damaged mitochondria that have deleterious mitochondrial DNA (mtDNA) mutations can regain their physiological function by fusion (Figure 2(b)).

### 2.3. Mitophagy

Mitophagy is the intracellular process whereby damaged or excess mitochondria are degraded and cleared, but a baseline level of mitophagy exists that helps maintain cellular homeostasis under physiological conditions. However, the decline in mitochondrial function with age leads to an accumulation of damaged mitochondria, which accelerates the aging process [19]. In addition, pathological conditions may involve continuous stimulation, causing a dysfunction in mitophagy, which accelerates cell death [20]. Since its discovery, mitophagy has been intensively studied, and there have been substantial advances in our knowledge of the molecular mechanisms involved. Briefly, the process of mitophagy begins with the separation of damaged mitochondria from the mitochondrial network and is followed by the envelopment of the injured organelles by isolated membranes (also known as phagophores) and their transport to lysosomes for degradation. According to the methods by which a phagophore bind to the damaged mitochondria, the regulation of mitophagy can be accomplished in two ways: by ubiquitin, involving phosphatase and tensin homolog-putative kinase 1 (PINK1) and the E3 ubiquitin ligase Parkin, or through receptor-mediated regulation, involving outer mitochondria membrane (OMM) proteins such as BCL2 interacting protein 3 (BNIP3), BNIP3-like (BNIP3L/NIX), and FUN14 domain containing 1 (FUNDC1) [21] (Figure 2(c)). There is an intimate relationship between mitochondrial dynamics and mitophagy. The separation of damaged mitochondria from the mitochondrial network into isolated components is considered to be a prerequisite for triggering mitophagy [22]. In turn, mitophagy regulates mitochondrial dynamics through the proteasomal degradation of the fusion protein Mfn2 and mitochondrial rho GTPase 1 (Miro) by Parkin [23].

### 2.4. Mitochondrial Biogenesis

Despite mitophagy having beneficial effects in terms of the removal of damaged mitochondria and the promotion of cell survival, it may also cause a decrease in the number of mitochondria, resulting in an impairment in mitochondria function. To maintain the mitochondria in a steady state, organisms generate new mitochondria to replace lost organelles through mitochondrial biogenesis. A balance between mitophagy and mitochondrial biogenesis (mitochondrial turnover) is required to optimize mitochondrial metabolism, and an imbalance in pathological states accelerates disease progression and limits damage repair [24].

Mitochondrial biogenesis is the formation of new organelles from existing mitochondria through division and proliferation [25]. Peroxisome proliferator-activated receptor gamma coactivator 1 alpha (PGC-1*α*)/nuclear respiratory factor 1 (Nrf1)/transcription factor A of mitochondria (TFAM) is the classical regulatory pathway of mitochondrial biogenesis, but the discovery of the ARE in the Nrf1 promoter implied that Nrf2 is an upstream transcriptional regulator of Nrf1 [26]. In addition, PGC-1*α* increases Nrf2 transcriptional activity through a direct interaction with Nrf2 [27], while Nrf2 increases PGC-1*α* expression by binding to the ARE on the PGC-1*α* gene [28]. In summary, Nrf2 enhances mitochondrial biogenesis through a positive feedback mechanism involving these regulatory factors (Figure 2(c)).

## 3. Dysregulation of Mitochondrial Homeostasis in Kidney Disease

### 3.1. Oxidative Stress in Kidney Disease

The kidney is characterized by high oxygen consumption and rapid metabolism and therefore is subject to significant oxidative stress and severe oxidative damage when under stress or in disease. Oxidative stress is a pathophysiological state that is characterized by the generation of large amounts of ROS and oxidative byproducts, which is caused by an augmentation of oxidase activity and a deficiency of antioxidants, which results in oxidative damage to macromolecules and the upregulation of other destructive processes, such as mitochondrial injury, inflammation, apoptosis, and fibrosis. Mitochondrial dysfunction is the principal cause of oxidative stress in kidney disease [29]. In addition, common comorbidities of kidney disease, such as diabetes, dyslipidemia, hypertension, and aging, are also important inducers of oxidative stress. The high concentrations of H_2_O_2_ that are present in acute or chronic kidney injury play a key role in the promotion of renal injury via an increase in NADPH oxidase (NOX)4 expression and a reduction in catalase activity [30–33].

NOX4, previously described as “Renox,” is the oxidase that is most responsible for the oxidative burst in renal disease [32]. Meng et al. [34] utilized in vitro and in vivo models of cisplatin-induced AKI to elucidate the role of NOX4 during AKI and showed that NOX4 protein and mRNA levels are upregulated by cisplatin. Furthermore, the inhibition of NOX4 expression by NOX4 shRNA or apocynin (a NOX inhibitor) reduced programmed cell death and renal inflammation, while the overexpression of NOX4 had the opposite effects. Moreover, the antioxidant N-acetyl-L-cysteine reduced NOX4 overexpression-induced cellular injury. Thus, NOX4 plays a crucial role in the promotion of AKI induced by cisplatin. Similarly, a detrimental effect of NOX4 activation and a renoprotective effect of NOX4 clearance have been shown in renal ischemia/reperfusion injury [35] and diabetic nephropathy [36].

It is well known that functional defects in the endogenous antioxidant system play an important role in oxidative stress and oxidative damage, but Kasuno et al. [37] have provided evidence for additional roles of antioxidant enzymes in kidney disease. This group showed that thioredoxin 1 (TRX1), a significant redox-regulatory protein, is secreted by cells that are under oxidative stress and that extracellular TRX1 has anti-inflammatory and antiapoptotic roles in the circulation. In addition, the urinary TRX1 concentrations of patients with AKI are markedly higher than those of patients with CKD or normal people, and as a result, the use of urinary TRX1 as a diagnostic marker for AKI has been suggested. Finally, an increase in oxidative stress in inflammation and fibrosis plays a significant role in the development of kidney injury.

The transcription factor nuclear factor-kappa B (NF-*κ*B), a key regulator of the inflammatory response, is activated by ROS, and the inflammatory injury caused by NF-*κ*B in turn worsens the oxidative stress. Thus, oxidative stress and inflammation exacerbate one another, causing a worsening of the renal damage [38]. Transforming growth factor *β*1 (TGF-*β*1) is a key profibrotic factor that has a similar effect to NF-*κ*B by worsening fibrosis via the formation of a signaling feedback loop with ROS: ROS upregulates TGF-*β*1, and TGF-*β*1 in turn amplifies oxidative stress by activating NOX4 [39]. More recently, Lu et al. have reviewed the role of the uremic toxin indoxyl sulfate in the redox imbalance that increases the incidences of pathological complications, cardiovascular disease, and mortality associated with CKD [40].

### 3.2. Kidney Disease Is Characterized by Greater Fission and Less Fusion of Mitochondria

Mitochondrial fragmentation is the typical pathological feature of acute and chronic renal injury, which implies that there is an imbalance in the fission and fusion of mitochondria in kidney disease. Abnormal activation of Drp1 and the inhibition of Mfn2 are the principal causes of excess mitochondrial fission in disease states. This mitochondrial fission induces apoptosis by activating the proapoptotic protein Bax, which causes further kidney damage [41]. Brooks et al. [42] evaluated the effects of mitochondrial dynamics on AKI using models of ischemic and cisplatin-induced AKI in rat proximal tubular cells and primary proximal tubular cells isolated from C57BL/6 mice. They showed that the substantial mitochondrial fragmentation during the early stages of AKI is associated with high Drp1 activity and that inhibition of Drp1 using genetic or pharmacological approaches significantly reduces cytochrome C release, caspase activation, and tubular cell apoptosis. In addition, using in vivo models of renal ischemia/reperfusion injury, Perry et al. [43] showed that Drp1 deficiency increases the expression of Ki67, an antigen associated with proliferating cells, and PGC-1*α*, which reduces plasma creatinine concentration and ameliorates tubular atrophy, implying that the inhibition of Drp1 promotes damage repair in the kidney. Furthermore, Wang et al. [44] used a unilateral ureteral obstruction surgery-induced model of fibrosis and normal rat kidney fibroblast cells (NRK-49F) to show that the excess mitochondrial fission that is mediated by phosphorylated Drp1 participates in the transformation of fibroblasts into myofibroblasts in kidney injury by increasing fibrin levels and promoting glycolysis. Thus, excess mitochondrial fission contributes to the progression of chronic fibrosis. Moreover, Ayanga et al. [45] showed that the knockout of Drp1 protein in the podocytes of diabetic mice has a renoprotective effect by reducing proteinuria, improving mitochondrial function, and reducing mitochondrial destruction and podocyte injury.

In disease, the negative regulation of Mfn2 proteins not only increases mitochondrial fission but also impairs mitochondrial fusion, which has substantial effects on the energy and nutrient metabolism of individual mitochondria [46]. In addition, Gall et al. [47] demonstrated that Mfn2 deficiency in proximal tubular epithelial cells substantially increases cellular apoptosis in states of stress, which are characterized by ATP depletion. Furthermore, Brooks et al. [48] showed that the apoptotic protein Bax is activated by mitochondrial fission and exacerbates renal injury. Using an in vitro model of cisplatin- and azide-induced AKI, they found that the overexpression of mitofusins or the inhibition of Drp1 reduces cytochrome C release from mitochondria and apoptosis by blocking the insertion of Bax into the OMM. Moreover, the inhibition of mitofusins has been shown to increase cisplatin-induced Bax activation, the release of cytochrome C, and apoptosis. In summary, excess mitochondrial fission promotes kidney damage in both acute and chronic kidney injury, which implies that the inhibition of excess mitochondrial fission may represent a therapeutic target in kidney disease.

### 3.3. Kidney Disease Is Characterized by Dysfunctional Mitophagy

To meet its high energy demand, the kidney is rich in mitochondria. However, mitochondria are prone to mutations in their DNA and organelle damage. Therefore, to maintain healthy mitochondrial homeostasis, it is necessary to remove damaged mitochondria by mitophagy. Lei et al. [49] showed that an inducer of mitophagy, rapamycin, ameliorates injury in HK-2 cells, whereas 3-methyladenine, an inhibitor of mitophagy, worsens the injury. However, Zhao et al. [50] demonstrated both in vitro and in vivo that glycoprotein stanniocalcin-1 (STC1) (a known mitochondrial-targeted antioxidant) inhibits renal injury in contrast agent-induced AKI, but that this is accompanied by a decrease in mitophagy, which is inconsistent with the previous findings. The authors hypothesized that STC1 pretreatment results in a low level of mitophagy, which reflects the effect of STC1 on mitochondrial injury, according to the principle that mitophagy is initiated by mitochondrial injury. Thus, in summary, an increase in mitophagy helps to mediate the effective clearance of damaged mitochondria during kidney injury.

A number of signaling pathways that regulate mitophagy, including the PINK1/Parkin, BNIP3, and Drp1 pathways, have been shown to promote renal injury repair in AKI. Firstly, a deficiency of PINK1 and Parkin has been shown to aggravate tubular injury in mice by inhibiting mitophagy and accelerating mitochondrial damage [51]. In addition, PINK1/Parkin-mediated mitophagy has been demonstrated to protect against AKI induced by cisplatin, sepsis, or a contrast agent [52–54]. Secondly, Tang et al. [55] have shown a renal protective effect of BNIP3-mediated mitophagy in mouse models of renal ischemia/reperfusion injury. They showed that the shRNA-induced silencing of BNIP3 in cultured renal tubular cells reduces mitophagy and potentiates cell death, and that BNIP3 knockout in mice worsens renal dysfunction and tissue damage, which confirms that BNIP3 has an important role in mitophagy and cell survival. Lastly, Li et al. [56] showed that pretreatment with mdivi-1, an inhibitor of Drp1, impairs mitophagy and worsens renal dysfunction and tubular cell apoptosis in a model of renal ischemia/reperfusion injury. Previous studies have also shown that impaired mitophagy and the accumulation of damaged mitochondria accelerate the aging process and the progression of age-related kidney disease [57]. Moreover, mitophagy disorders in podocytes contribute to the progression of diabetic nephropathy. Fang et al. [58] showed that diabetes in vivo and a high-glucose concentration in vitro are associated with defects in autophagy in podocytes, which promote cellular injury and accelerate the progression of diabetic nephropathy. Thereafter, using streptozotocin-induced diabetic mice and a mouse podocyte line cultured in high-glucose medium, Li et al. [59] showed that forkhead-box class O1 (FoxO1) promotes podocyte injury repair and delays the progression of diabetic nephropathy by inducing PINK1/Parkin-mediated mitophagy. Therefore, in summary, the promotion of mitophagy in acute and chronic renal injury may be of therapeutic benefit.

### 3.4. Deficiency of Mitochondrial Biogenesis in Kidney Disease

Mitochondrial biogenesis is critical for the repair of cellular damage in AKI. PGC-1*α* is the most important transcriptional regulator of mitochondrial biogenesis and is therefore often studied during research into the role of mitochondrial biogenesis in renal damage repair. For example, Rasbach and Schnellmann [60] treated renal proximal tubular cells isolated from rabbits using T-butylhydroperoxide to induce the type of mitochondrial dysfunction that is associated with ischemia/reperfusion injury and found that sublethal cell damage was repaired within 6 days. During this process, the expression of PGC-1*α* increased significantly within 24 hours and was maintained at a high level until recovery was complete, which suggests that PGC-1*α*-mediated mitochondrial biogenesis ameliorates kidney injury and promotes damage repair. In subsequent work, this group further characterized the role of PGC-1*α* in the promotion of damage repair by overexpressing PGC-1*α* prior to exposure to oxidants [5]. They found that this did not preserve mitochondrial function but instead potentiated dysfunction and cell death, which could be explained by an increase in mitochondrial biogenesis during the injury period creating large numbers of damaged mitochondria, which would exacerbate the injury. However, increasing the expression of PGC-1*α* after oxidant exposure dramatically accelerated the restoration of the cells, probably because at this stage there was high demand for regenerating mitochondria to facilitate the repair process.

Funk an Schnellmann [61] studied the role of mitochondrial biogenesis in animal models of myoglobinuric AKI and ischemic AKI. In both models, the structure and function of the renal tubules were impaired, but there was partial recovery of glomerular function. The expression of PGC-1*α* was high during the early stages of the insult and remained high throughout the repair process. However, the expression of mitochondrial respiratory proteins was very low during the early stages and did not recover until the later stages of repair. Thus, PGC-1*α* expression increases rapidly after the initiation of AKI, but its transcriptional activity is inhibited by ongoing injurious stimulation, which leads to mitochondrial defects and poor damage repair. By contrast, the expression of PGC-1*α* is downregulated in folic acid-induced and sepsis-associated AKI, and the overexpression of PGC-1*α* ameliorates the deleterious effects of both insults [62, 63]. Furthermore, Fontecha-Barriuso et al. [64] demonstrated that PGC-1*α* deficiency exacerbates cell injury by inhibiting mitochondrial biogenesis and increasing inflammation in a mouse model of folic acid-induced AKI.

In summary, even though the levels of PGC-1*α* expression differ according to the type of AKI, increasing its activity assists damage repair in AKI. However, the effects of mitochondrial biogenesis in chronic renal diseases are not universal. The overexpression or pharmacological activation of PPAR*γ* is an effective means of upregulating PGC-1*α*. In db/db diabetic mice, mouse podocytes, and renal mesangial cells cultured in high-glucose medium, the PPAR*γ* agonist rosiglitazone has renoprotective effects, ameliorating oxidative stress, glomerulosclerosis, and tubulointerstitial fibrosis by increasing PGC-1*α* expression [65]. Another study also showed that renal fibrosis in diabetic rats is inhibited by the genetic or pharmacological activation of PPAR*γ* [66]. Although PGC-1*α* has been reported to have a protective effect in most studies of CKD, Li et al. [7] showed that excessive PGC-1*α* increases proteinuria and impairs renal function in diabetic mice with podocyte-specific overexpression of PGC-1*α*. Furthermore, PGC-1*α* overexpression has also been shown to cause loss of sarcomeric structure and a dilated cardiomyopathy in cardiomyocytes [67]. Although it is not fully understood, the mechanism of the adverse effects of PGC-1*α* in chronic diseases may involve the promotion of angiogenesis after ischemia because this process aggravates ischemic and hypoxic damage to intrinsic cells in kidney, which promotes fibrosis [68].

## 4. Role of Nrf2 Activation in the Regulation of Mitochondrial Homeostasis in Kidney Disease

### 4.1. Ischemia/Reperfusion-Induced AKI

Renal ischemia/reperfusion injury (RIRI) is a serious complication of critical illness and surgery that is associated with a poor prognosis for the primary disease and a significant possibility of AKI-associated mortality [69]. The pathogenesis of RIRI involves ischemic/hypoxic injury to proximal tubular and endothelial cells [70]. Ischemia-associated hypoxia leads to renal damage and microvascular dysfunction through inflammatory and oxidative stress cascades, leading to hypoxic injury and ROS accumulation in the kidney. During this process, oxygen supply and demand are severely affected by the lower blood flow to the renal medulla, which affects solute transport activity. Subsequently, reperfusion is associated with a sustained increase in solute transport demand that cannot be facilitated by the level of reoxygenation, which worsens the oxygen deficit, leading to further ROS accumulation and kidney injury [71]. The pathophysiological mechanisms of RIRI also include mitochondrial injury, apoptosis, necrosis, and inflammation, in which mitochondrial injury plays a central role [72]. The severe oxygen deficit and oxidative stress that are induced by hypoxia and reoxygenation severely affect mitochondrial homeostasis. Nrf2 is the most important component of the antioxidant defense system and has been shown to play a crucial protective role in RIRI. Leonard et al. [73] showed for the first time using microarray analysis that the expression of Nrf2 and Nrf2-dependent antioxidative genes is induced as an adaptive mechanism to reduce renal cellular injury in a model of ischemia/reperfusion. They also showed that the deletion of Nrf2 increases the susceptibility of mice to RIRI and that antioxidant treatment protects against kidney damage, thereby confirming that Nrf2 ameliorates RIRI by inducing an antioxidant response [74]. Zhang et al. [75] studied the effects of simvastatin on RIRI in rats and found that it has a protective role that is mediated by the activation of Nrf2/HO-1 and a restoration of redox homeostasis. Furthermore, Nezu et al. [76] demonstrated that Nrf2 protects against RIRI-induced renal damage using Nrf2 and Keap1 knockdown mice, showing that Nrf2 has positive effects on redox status, mitochondrial biogenesis, and cell proliferation. In addition, they determined whether the timing of activation of Nrf2 is important by pharmacologically activating Nrf2 during the early or late stages of the insult. They found that Nrf2 activation during the early stages, but not the late stages, ameliorates renal tubular injury. Thus, the early activation of Nrf2 appears to be important in slowing the progression of renal tubular injury.

### 4.2. Septic AKI

Sepsis is a clinical syndrome that is caused by bacterial infection and the resulting systemic inflammatory response and that often results in multiple organ failure, including renal failure, which is referred to as septic acute kidney injury (SAKI). In patients in the intensive care unit, SAKI is a serious complication that is characterized by high morbidity, high mortality, and a poor prognosis [77]. In such patients, antibacterial treatment and hemodialysis to treat the uremia do not reduce the short-term incidence of mortality or the long-term incidence of CKD [78]. Therefore, it is important to further elucidate the pathogenesis of SAKI to identify more effective treatments.

A study by Takasu et al. [79] showed that there is a discordance between renal dysfunction and structural damage in patients with SAKI. They found that the majority of patients with sepsis had tubular injury, but that these injuries were relatively minor, such as focal lesions or small areas of necrosis, while large areas of tubular injury or tubular necrosis were rare, which was inconsistent with the severe renal dysfunction in these patients. Furthermore, the ultrastructural features of the injury were principally mitochondrial swelling, lysosomal expansion, and greater autophagy. Thus, the mild renal tissue injury in patients with SAKI cannot explain the severe renal dysfunction. However, the characteristics of the mitochondrial injury identified in this study are consistent with the previously reported improvements in SAKI that can be achieved through an upregulation of mitochondrial biogenesis [62], and the results of both of these studies imply that mitochondrial dysfunction plays an important role in the pathogenesis of SAKI.

The effects of Nrf2 in mitochondrial homeostasis and its protective effect in a model of SAKI prompted further exploration of the role of Nrf2 in mitochondrial homeostasis in SAKI and mechanism involved. Multiple experimental studies have shown that Nrf2 ameliorates SAKI by regulating mitochondrial redox homeostasis and reducing oxidative stress in cells and mitochondria [80–82]. The introduction of human glomerular cord blood mononuclear cells into a rat model of SAKI had a renoprotective effect by activating Nrf2, thereby promoting mitophagy [83], and this represents a promising new therapeutic approach. A study by Liu et al. [84] showed that the activation of Nrf2 by the antioxidant procyanidin B2 ameliorates renal injury and tubular cell apoptosis in mice with SAKI by improving mitochondrial dynamics and increasing mitophagy. Notably, Gonzalez et al. [4] demonstrated that the early recovery of mitochondrial dynamics is important in the resolution of septic organ failure by studying two models of sepsis: endotoxemia induced by lipopolysaccharide (LPS) and cecal ligation and puncture (CLP). They showed that the mortality rate associated with CLP was much higher than that associated with endotoxemia, and consistent with this, the recovery of the mitochondria in CLP was worse than that in endotoxemia. Furthermore, in endotoxemia, an impairment in mitochondrial dynamics developed soon after the onset, but this began to resolve after 24 hours, whereas mitochondrial biogenesis did not commence within 24 hours. Instead, the severe damage to the mtDNA had begun to recover after 48 h. Therefore, they concluded that successful recovery from septic organ failure depends on the early restoration of mitochondrial dynamics, rather than of mitochondrial biogenesis, and this is corroborated by the fact that pretreatment with mdivi-1 (a Drp1 inhibitor) significantly ameliorates mitochondrial dysfunction and apoptosis in CLP.

### 4.3. Drug-Induced AKI

In clinical practice, many of the drugs that are excreted by the kidney, such as antineoplastic drugs, antibiotics, and contrast agents, can cause acute renal dysfunction, a condition known as drug-induced AKI. Perazella and Luciano [85] prepared a detailed summary of the drugs that can induce renal injury, including the hemodynamic, vascular, glomerular, and tubulointerstitial types. In most cases, symptomatic treatment, such as the withdrawal of the nephrotoxic drug and administration of a corticosteroid, is sufficient to permit full recovery from drug-induced AKI. However, successful management of the therapeutic and nephrotoxic effects of such nephrotoxic drugs when they cannot be withdrawn remains a challenge for physicians, and this is an important reason why researchers continue their efforts to understand the pathogenesis of drug-induced AKI and develop specific renoprotective drugs. As in other types of kidney injury, mitochondrial dysfunction and low Nrf2 activity are involved in the pathogenesis of drug-induced AKI. Cisplatin is a widely used and effective chemotherapeutic agent, but it has many side effects, including nephrotoxicity, which is a serious complication associated with a poor prognosis [86]. Cisplatin is a positive electrophilic reagent in cells, and in particular in the tubular epithelial cells of the kidney, such that it specifically accumulates in the negatively charged mitochondria, which can induce large areas of mitochondrial damage, resulting in extensive mitophagy and potentially a malignant event [87]. Thus, in cisplatin-induced renal injury, strategies aimed at ameliorating mitochondrial damage and suppressing excessive mitophagy contribute to the rescue of normal mitochondria and the preservation of cellular function. Nrf2 has been shown to inhibit cytochrome C release and apoptosis in cisplatin-induced apoptotic HK-2 cells, which implies that it plays an important role in maintaining mitochondrial structural integrity and function [88]. Furthermore, Nrf2 activation has been shown to be protective in the kidney, by regulating the redox homeostasis in the mitochondria, in an in vivo model of cisplatin-induced AKI [89]. In contrast to cisplatin-induced AKI, greater mitophagy was shown to ameliorate renal injury in contrast agent-induced AKI, whereas lower mitophagy exacerbated the injury [49]. In another study, it was shown that drug preconditioning of STC1 protects against contrast-induced AKI by modulating mitochondrial dynamics and reducing mitochondrial damage, such that there is less mitophagy [50]. As in the situation outlined above, these two findings are not contradictory, and the latter highlights the key role of Nrf2 activation in the regulation of mitochondrial homeostasis and the protection of mitochondria, as well as the beneficial effects of early treatment for the preservation of mitochondria and the promotion of recovery from kidney injury.

### 4.4. Diabetic Kidney Disease (DKD)

DKD, also known as diabetic nephropathy, is the most studied disease with respect to the protective effects of Nrf2 activation in CKD. DKD is a common and serious complication of diabetes, as well as a major cause of CKD and ESRD, and it is characterized by poor renal function, massive proteinuria, and progressive renal fibrosis. The early pathological features of DKD are glomerular injury with mesangial dilation, basement membrane thickening, and podocyte loss; and these are followed by damage to renal tubules and the interstitium, reflected in tubular basement membrane thickening, tubular atrophy, interstitial fibrosis, and arteriosclerosis [90]. The pathophysiological mechanism of DKD involves hyperglycemia, damage to intrinsic renal cells, and abnormal hemodynamics. The damage to the glomerular filtration barrier that is caused by podocyte injury results in large amounts of protein entering the urine in DKD. In addition, damage to mesangial and endothelial cells causes hemodynamic abnormalities that include dilation of afferent arterioles and renal vasoconstriction, as well as proteinuria and abnormal glucose metabolism, which increases oxidative stress and mitochondrial damage in renal cells [91].

Nrf2 has been shown to play a protective role in experimental studies of DKD, mediated by its antioxidant effects and effects on mitochondrial homeostasis. A study by Jiang et al. [92] showed that the expression of Nrf2 in kidney biopsies from patients with DKD is higher than that in healthy individuals. Furthermore, in vitro and in vivo studies of streptomycin-induced DKD have shown that Nrf2 significantly reduces renal structural damage and functional impairment by ameliorating oxidative stress and fibrosis. However, it should be noted that the adaptive antioxidative effects of Nrf2 cannot prevent the progression of DKD in humans, whereas Nrf2 activation by genetic or pharmacological means is protective in mice. Because of the contrast between the delayed activation of Nrf2 in humans and the effects of activation in experimental studies, it seems that the early activation of Nrf2 may be more effective at preserving renal tubules and delaying renal fibrosis than its late activation. Indeed, Xiao et al. [93] demonstrated that early treatment of db/db mice or the pretreatment of high glucose-treated renal tubular epithelial cells with the mitochondrial-targeted antioxidant MitoQ improved damage repair in renal tubular cells by ameliorating mitochondrial oxidative stress, improving mitochondrial dynamics, and increasing mitophagy via activation of the Nrf2/PINK1 pathway.

Bardoxolone methyl (BARD), an activator of Nrf2, was first tested in a phase I cancer trial, in which Nrf2 activation was found to significantly improve estimated glomerular filtration rate (eGFR) [94]. However, in subsequent clinical trials conducted in patients with type 2 diabetes mellitus and stage 4 CKD (the phase 3 BEACON study), and despite an improvement in eGFR, BARD was associated with greater proteinuria and serious cardiovascular events, which led to premature termination of the trial [95]. Subsequently, Rush et al. [96] also showed that Nrf2 activation in mice can aggravate CKD. To determine whether the use of BARD in patients with DKD is associated with the maintenance of eGFR, the phase 2 TSUBAKI study was performed in patients with diabetes and stage 3 CKD [97], and the preliminary findings were that BARD significantly improved eGFR without increasing the incidence of cardiovascular events. These studies suggest that Nrf2 activation may be an effective and safe intervention during the early stages of CKD but may increase the risk of adverse events during the late stages. Thus, Nrf2 activation has therapeutic potential in patients with CKD, but the timing of administration may be the key to its successful use.

## 5. Conclusions

In general, the development of AKI can be divided into three stages: early injury, worsening of the injury, and recovery from the injury [61]. With the evolution of the pathophysiological environment during the stages of AKI, the mitochondrial events that affect the progression of recovery from the disease change. In the early stage of AKI, activation of the fission and impairment of the fusion of mitochondria result in severe structural damage and functional deficits, which lead to impairments in mitochondrial energy generation and apoptosis. Therefore, improvements in mitochondrial dynamics during the early stage of injury play a decisive role in recovery [4]. Subsequently, mitophagy is upregulated as the number of damaged and defective mitochondria increases. At this stage, redox status, mitochondrial dynamics, and mitochondrial biogenesis influence the progression of the injury [76, 84]. Conversely, the upregulation of mitochondrial biogenesis may be deleterious during the early stage, with an increase in the number of immature mitochondria resulting in larger numbers of damaged mitochondria, thereby exacerbating the defects [5]. One thing that is certain is that mitochondrial biogenesis has a decisive protective role during recovery from injury [6].

**Figure 3:**
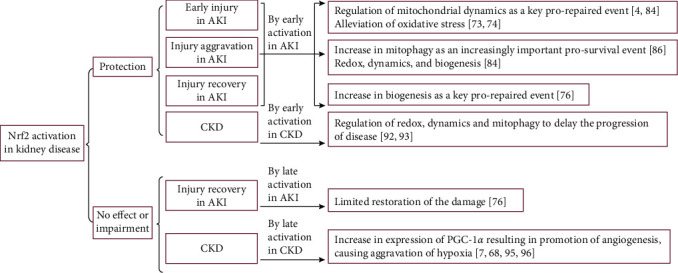
The effects of Nrf2 activation on mitochondrial homeostasis in kidney disease, according to the timing of activation. Nrf2 activation has various effects, including protection, impairment, and no effect during the early injury stage, worsening of the injury, and recovery from AKI and CKD, depending on the timing of activation (early or later activation in AKI or CKD).

Appropriate regulation of redox, mitochondrial dynamics, and mitophagy has been shown to be important in CKD [92, 93]. However, although protective effects of mitochondrial biogenesis have been identified, it has also been shown to be associated with greater fibrosis in DKD [7]. Furthermore, the resultant proangiogenic effect in the chronic phase is considered to cause a worsening of hypoxia in renal intrinsic cells [68]. It should be noted that early Nrf2 activation has been shown to have protective effects in a variety of models of AKI and CKD that are closely related to improvements in mitochondrial homeostasis, and late activation of Nrf2 has been shown to be associated with severe adverse effects in clinical trials conducted in patients with late-stage CKD (Figure 3).

On the basis that mitochondrial homeostasis adapts to the disease state, and the evidence that has accumulated from numerous studies, we propose that Nrf2, which regulates mitochondrial homeostasis, may have a stronger protective effect in AKI than in CKD. Thus, Nrf2 activation has therapeutic potential in kidney disease, and especially in AKI, but this must be evaluated in further clinical trials. Moreover, the mechanisms of the adverse reactions to an Nrf2 activator in patients with CKD should be clarified, in order to guide its clinical use. Interestingly, Nrf2 activators, flavonoids, have been shown the dual effects in cancer treatment, possibly due to the failure of the administered concentration to reach the effective inhibitory concentration, which provides useful ideas for Nrf2 activators to cause toxicity [98].
